# Trephining: Its Indications, Technique, after Treatment, and Sequelæ

**Published:** 1899-09

**Authors:** L. A. Merillat

**Affiliations:** Secretary of M’killip Veterinary College, Chicago


### DEPARTMENT OF SURGERY.

By L. A. Merillat, V.S.,
SECRETARY OF M'KILLIP VETERINARY COLLEGE, CHICAGO.

ASSISTED BY

W. E. A. Wyman, M.D.V., V.S.,

MILWAUKEE, WIS.

J. A. Sloan, B.Sc., M.D.V.,

SAN FRANCISCO, CAL.

Dr. Robert Jay, M.D.V.,
WEST LIBERTY, IA.

### Trephining: Its Indications, Technique, After-treatment, and Sequelæ.

(Continued from page 441.)

Technique—*Continued.* Granting now that the animal is secured and the seat of operation selected in the manner described in preceding chapters, the next step will consist of making the openings. Although this is a simple procedure, there are, however, a number of points worthy of discussion, having in mind the expediency of always being governed by set rules, even in the execution of the minutest details of surgical technique.

*Disinfection of the Operating Field.* The hair should be clipped from the entire nasal arch and the surface energetically scrubbed with soap and water, then washed for a few minutes with a mercuric-chloride solution (1 : 500). It has been said by recent writers on antiseptics that this solution is unnecessarily strong, if not dangerous. I have, however, never observed the least evidence of intoxication from such a solution, and, having had excellent results, I would reluctantly discard its use in this concentration for the purpose above mentioned, as well as for the disinfection of the unbroken skin generally. "Individual susceptibility" to mercury, either general or local, so frequently referred to in human medicine, has never been recognized in veterinary practice.

*Opening the Skull.* A crude method consists of simply removing the skin and then at once completing the opening with a trephine without further ceremony. A more circumspect and proper method, however, is to deal carefully with each structure as reached, viz.:

1. The skin: In dealing with the integument the operator should limit the cutting to the skin itself, thus exposing to view the subcutaneous and muscular tissue with but little hemorrhage. If a deep incision is made at once there is always troublesome hemorrhage, which cannot be arrested until the operation has proceeded further. The shape of the incision is also important. Some have recommended a crescent incision, so that the flap thus made can be replaced by sutures after the operation is complete; while others advise the removal of a circular piece the size of the trephine. The former can hardly be regarded as faultless, because the openings must be continually utilized in the after-treatment, and if the suturing is put off until the after-treatment is completed the flap of skin will be too shrunken to be replaced. The latter is objectionable, because too much skin is lost, and a scar will result. The aim should be to make an incision that will leave but little scar and at the same time permit free access to the cavity for the purpose of irrigation. This is done by removing an elliptical segment, the long diameter of which is no longer than that of the trephine and the short diameter from 1 to 2 cm., according to the size of the opening desired. Such an incision can be dilated to admit any size instrument.

2. The subcutaneous and muscular: Especially in the anterior facial region the muscular tissue is thick, and, therefore, requires careful removal. A circular piece must be removed so as to entirely clear the trephine, otherwise it will be torn and cause unnecessary injury. The hemorrhage, which is always considerable, must be completely arrested before proceeding further.

3. The periosteum: The object of removing the periosteum is to prevent it from being torn by the trephine. The saw of a veterinary trephine is usually dull, and will tear this tough membrane some distance around the opening unless some provision is made to prevent it. Such tearing is not ordinarily serious, but in isolated cases may prove to be a cause for more or less necrosis. Fistulæ and tardy healing of the opening can usually be traced to this source. A sharp curette, a periosteotome, or a common scalpel may be used in removing it.

4. The bone: The bone is removed with a circular trephine, and when the opening must be enlarged, or a special shaped one is required, a bone-chisel is used to complete the work. In starting the circular trephine the centre gimlet should project beyond the level of the saw-edge until some headway is made into the bone; then the gimlet should be withdrawn and the remainder of the sawing done so carefully that the mucous membrane will not be molested when the section is removed.

5. The mucous membrane: This is then removed with a forceps and scalpel, and the resulting hemorrhage arrested by pressure.

This simple routine should be carefully carried out in all trephining operations. There is then no blood to mar the effect, and the result is a neat, satisfactory procedure; but from this point the technique must vary according to the indication for which the operation is being performed, viz.:

*To Empty the Sinuses.* If the operation is undertaken with this intention the patient is permitted to regain the standing posture as soon as the openings are made, and the contents of the sinuses—usually desiccated pus—are carefully dislodged by syringing small quantities of antiseptic fluid, with a long-piped syringe, into every recess. The openings having been advantageously located, every point can be reached. This done the cavities are irrigated with a fountain syringe (a small hose fastened to a hole at the bottom of a pail makes a very useful fountain syringe for veterinary purposes). The operation is completed by plugging the openings with cotton or oakum disinfected with mercuric-chloride solution and then covering the field with a stiff iodoform ointment to exclude external influences until the after-treatment is begun. (For after-treatment see future chapters.)

*To Remove a Tooth.* The necessary instruments for this purpose are: A punch, slightly curved and bell-shaped at the distal end and corrugated at the contact extremity, a heavy wooden mallet, and a mouth-speculum. The speculum is adjusted and opened as wide as possible. The surgeon places one hand into the mouth and presses a finger tightly against the offending tooth, and with the other he directs the punch. The assistant is then instructed to apply the mallet gently, until the impression of the blow is felt by the hand in the mouth, and then forcibly until it is driven from its cavity. To direct the punch with one hand and wield the mallet with the other is not advisable, as in this position there is less assurance that the punch is being properly directed.

L. A. Merillat, V.S.

(To be continued.)

